# Supplementary material for: Exploring the Lifetime Effect of Children on Wellbeing Using Two-Sample Mendelian Randomisation
Source: Genes (Basel). 2023 Mar 14;14(3):716. doi: 10.3390/genes14030716 (PMC10048211; doi:10.3390/genes14030716)
Supplement: Supplementary file 1 [file genes-14-00716-s001.zip › cwb sup mat.pdf]

Supplementary Methods

Questions asked in the UKB

Information on siblings was ascertained through three questions asked at baseline recruitment. Number of full sisters (UKB ID: 1883, OpenGWAS ID: ukb-b-5593) asked the question "How many sisters do you have? (Please include those who have died, and twin sisters. Do not include half-sisters, step-sisters or adopted sisters)". Number of full brothers (UKB ID: 1873, OpenGWAS ID: ukb-b-4263) asked “How many brothers do you have? (Please include those who have died, and twin brothers. Do not include half-brothers, step-brothers or adopted brothers)"; and the number of older siblings (UKB ID: 5057, OpenGWAS ID: ukb-b-1997) "How many OLDER brothers/sisters do you have? (Please include those who have died, and twins. Do not include half-, step- or adopted brothers and sisters)". Hair colour (UKB ID: 1747, Open GWAS IDs: ukb-d-1747_5, ukb-d-1747_4, ukb-d-1747_3, ukb-d-1747_1, ukb-d-1747_2, ukb-d-1747_6) was ascertained through a questionnaire asked at baseline assessment. Participants were asked: "What best describes your natural hair colour? (If your hair colour is grey, the colour before you went grey)". To measure general happiness (UKB ID: 20458, OpenGWAS ID: ukb-b-4062) participants were ask "In general how happy are you?" and could select options from “Extremely happy”, “very happy”, “moderately happy”, “moderately unhappy”, “very unhappy”, or “extremely unhappy”.

Genotyping

UKB: All UKB GWASs which were used in this study were conducted using the MRC-IEU UKB GWAS pipeline (1). A full description of the pipeline methods can be found elsewhere. In brief, after a standardised quality control process, and imputation using the UK10K Haplotype Reference Consortium, the summary statistics were created using a linear mixed model implemented in BOLT-LMM (2), adjusting for sex and SNP-chip. Further information about each GWAS, including the number of participants included for each measure, can be found at https://gwas.mrcieu.ac.uk/.

SSGAC: Genotyping and QC information about the included samples are described the Supplementary Material original publication (3). The participating cohorts were asked to adjust their GWASs for the first four principal components of the genetic relationship matrix, as well as sex, age, age squared, and study-specific covariates for batch/study cite effects where appropriate. The LDSC intercept for the GWAS is 1.0188 (SE = 0.0076) implying the presence of a very small amount of residual population structure.

WFC: The participating cohorts from the WFC ran GWASs using a within-sibship model. This model adjusts for the mean genotype of each participant’s sibling, and inflates the standard error to account for clustering. The model additionally adjusted for age, sex, and the first twenty principal components. The results of the individual studies were then meta-analysed using a fixed-effects model. More details on the GWAS methods, including the QC employed, can be found in the original publication (4). The LDSC intercept for the GWAS implied no evidence of residual population structure (b = 1.005, SE = 0.0063).

Triangulation and planned interpretation of sensitivity analyses and negative controls.

Pleiotropy. Pleiotropy can bias MR estimates in either direction. However, on the assumption that pleiotropy introduces the statistical properties described above, if it is present we would expect to see evidence of heterogeneity in the SNP effect, and possibly outliers in the leave-one-out analysis. The different sensitivity analyses are robust to different types of pleiotropy and therefore can behave differently from each other. For example, if the MR-Egger and/or MR-PRESSO sensitivity analyses produce very different estimates from the other sensitivity analyses then this could be an indication of a violation of the InSIDE assumption. We would therefore interpret the sensitivity analyses as indicating the presence of pleiotropy if either all produce very different estimates from each other, or a plurality of them produce a consistently different estimate from IVW as an indication of residual pleiotropy.

Residual confounding. If there is residual confounding due to population structure or passive gene-environment correlation, then we would expect to find consistent evidence in their respective negative control analysis. Because residual confounding can also bias MR estimates in either direction, we would then expect this to lead to the point estimates between the SSGAC and WFC GWAS to be different, although a small chance variation is to be expected because of the reduced precision in the within family GWAS.

Low power. If the power of the study can be improved by adding more weakly associated SNPs then we would expect that the 95% CI for the IVW estimate would be smaller when using a $p < 5 \times 10^{-6}$ threshold than the genome-wide significant one. However, interpreting any change in the point estimate after this sensitivity analysis can be difficult because the use of weaker instruments in a two-sample setting will create a (hopefully small) bias towards the null. In addition, because these SNPs have a weaker association, there is a greater risk that any association they do have is due to some type of confounding, which could bias estimates in either direction.

Software and Preregistration

MR analyses in this paper were run using the TwoSampleMR, MR-RAPS, MR-PRESSO, and meta R packages (5–8). The DAGs were drawn using DAGitty (9). All GWAS data was extracted from the MRC-IEU OpenGWAS platform (10).

This study was written in accordance with STROBE-MR (11), and was pre-registered at https://DOI.org/10.17605/OSF.IO/BTPH9. The quantitative assessment of whether samples were drawn from the same population was not part of our pre-registered analysis plan.

References

1. Ruth Mitchell E. MRC IEU UK Biobank GWAS pipeline version 2 [Internet]. data.bris. 2019 [cited 2022 Mar 12]. Available from: https://data.bris.ac.uk/data/dataset/pnoat8cxo0u52p6ynfaekeigi

2. Loh PR, Tucker G, Bulik-Sullivan BK, Vilhjálmsson BJ, Finucane HK, Salem RM, et al. Efficient Bayesian mixed-model analysis increases association power in large cohorts. Nat Genet. 2015 Mar;47(3):284–90.

3. Okbay A, Baselmans BML, De Neve JE, Turley P, Nivard MG, Fontana MA, et al. Genetic variants associated with subjective well-being, depressive symptoms, and neuroticism identified through genome-wide analyses. Nat Genet. 2016;48(6):624–33.

4. Howe LJ, Nivard MG, Morris TT, Hansen AF, Rasheed H, Cho Y, et al. Within-sibship GWAS improve estimates of direct genetic effects [Internet]. bioRxiv; 2021 [cited 2022 Mar 12]. p.

2021.03.05.433935. Available from: https://www.biorxiv.org/content/10.1101/2021.03.05.433935v1

5. Zhao Q, Wang J, Hemani G, Bowden J, Small DS. Statistical inference in two-sample summary-data Mendelian randomization using robust adjusted profile score. arXiv:180109652 [math, stat] [Internet]. 2018 Jan 29 [cited 2019 Apr 17]; Available from: http://arxiv.org/abs/1801.09652

6. Verbanck M, Chen CY, Neale B, Do R. Detection of widespread horizontal pleiotropy in causal relationships inferred from Mendelian randomization between complex traits and diseases. Nat Genet. 2018 May;50(5):693–8.

7. Hemani G, Zheng J, Elsworth B, Wade KH, Haberland V, Baird D, et al. The MR-Base platform supports systematic causal inference across the human phenome. eLife. 2018 May 30;7:e34408.

8. Schwarzer G, Carpenter JR, Rücker G. Meta-Analysis with R [Internet]. Cham: Springer International Publishing; 2015 [cited 2022 Apr 14]. (Use R!). Available from: http://link.springer.com/10.1007/978-3-319-21416-0

9. Textor J, van der Zander B, Gilthorpe MS, Liśkiewicz M, Ellison GT. Robust causal inference using directed acyclic graphs: the R package ‘dagitty.’ International Journal of Epidemiology. 2016 Dec 1;45(6):1887–94.

10. Elsworth B, Lyon M, Alexander T, Liu Y, Matthews P, Hallett J, et al. The MRC IEU OpenGWAS data infrastructure [Internet]. bioRxiv; 2020 [cited 2022 Mar 30]. p. 2020.08.10.244293. Available from: https://www.biorxiv.org/content/10.1101/2020.08.10.244293v1

11. Skrivankova VW, Richmond RC, Woolf BAR, Davies NM, Swanson SA, VanderWeele TJ, et al. Strengthening the reporting of observational studies in epidemiology using mendelian randomisation (STROBE-MR): explanation and elaboration. BMJ. 2021 Oct 26;375:n2233.
