# Supplementary material for: Exploring the Lifetime Effect of Children on Wellbeing Using Two-Sample Mendelian Randomisation
Source: Genes (Basel). 2023 Mar 14;14(3):716. doi: 10.3390/genes14030716 (PMC10048211; doi:10.3390/genes14030716)
Supplement: Supplementary file 1 [file genes-14-00716-s001.zip › cwb Supplementary Figure S1.pdf]

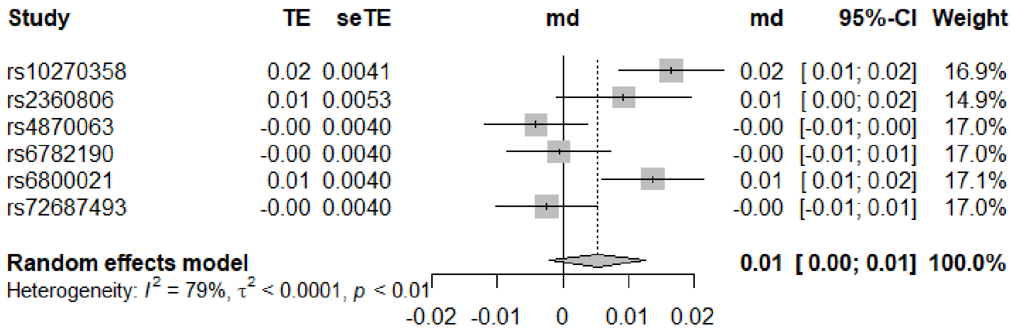

Supplementary Figure S1: Difference in SNP-outcome associations between the UKB and SSGAC when using a $5x10^{-8}$ p-value threshold.
