# Supplementary material for: Exploring the Lifetime Effect of Children on Wellbeing Using Two-Sample Mendelian Randomisation
Source: Genes (Basel). 2023 Mar 14;14(3):716. doi: 10.3390/genes14030716 (PMC10048211; doi:10.3390/genes14030716)
Supplement: Supplementary file 1 [file genes-14-00716-s001.zip › cwb Supplementary Figure S3.pdf]

Study	TE	seTE	md	95%-CI	Weight
rs10270358	0.02	0.0041	0.02	[0.01; 0.02]	2.2%
rs10787738	-0.00	0.0050	-0.00	[-0.01; 0.01]	1.8%
rs10965780	0.00	0.0040	0.00	[-0.01; 0.01]	2.2%
rs11038919	0.01	0.0052	0.01	[0.00; 0.02]	1.7%
rs11084855	0.01	0.0040	0.01	[0.00; 0.01]	2.2%
rs11165771	0.00	0.0053	0.00	[-0.01; 0.01]	1.7%
rs11638204	-0.00	0.0053	-0.00	[-0.01; 0.01]	1.7%
rs12203592	-0.01	0.0067	-0.01	[-0.02; 0.01]	1.2%
rs140120	-0.00	0.0039	-0.00	[-0.01; 0.01]	2.2%
rs1510400	0.01	0.0040	0.01	[0.00; 0.02]	2.2%
rs16990144	-0.01	0.0066	-0.01	[-0.02; 0.01]	1.3%
rs174549	-0.00	0.0041	-0.00	[-0.01; 0.00]	2.2%
rs17467404	-0.00	0.0041	-0.00	[-0.01; 0.01]	2.2%
rs199460	-0.01	0.0052	-0.01	[-0.02; 0.00]	1.7%
rs2015629	-0.00	0.0039	-0.00	[-0.01; 0.01]	2.3%
rs2077705	0.01	0.0050	0.01	[0.00; 0.02]	1.8%
rs2221757	-0.00	0.0039	-0.00	[-0.01; 0.01]	2.2%
rs2333545	0.01	0.0051	0.01	[0.00; 0.02]	1.7%
rs2356662	-0.01	0.0050	-0.01	[-0.02; 0.00]	1.8%
rs2360806	0.01	0.0053	0.01	[0.00; 0.02]	1.7%
rs2406734	-0.00	0.0040	-0.00	[-0.01; 0.00]	2.2%
rs2950924	-0.00	0.0040	-0.00	[-0.01; 0.01]	2.2%
rs3007233	-0.01	0.0039	-0.01	[-0.01; 0.00]	2.3%
rs341278	0.01	0.0062	0.01	[0.00; 0.02]	1.4%
rs36003933	-0.01	0.0050	-0.01	[-0.02; 0.00]	1.8%
rs4053614	-0.00	0.0062	-0.00	[-0.02; 0.01]	1.4%
rs4518438	-0.00	0.0039	-0.00	[-0.01; 0.01]	2.3%
rs4574383	0.00	0.0051	0.00	[-0.01; 0.01]	1.7%
rs4655026	0.01	0.0040	0.01	[0.00; 0.01]	2.2%
rs4870063	-0.00	0.0040	-0.00	[-0.01; 0.00]	2.2%
rs4986172	-0.00	0.0040	-0.00	[-0.01; 0.01]	2.2%
rs6085054	-0.00	0.0040	-0.00	[-0.01; 0.01]	2.2%
rs62146645	-0.01	0.0049	-0.01	[-0.02; 0.00]	1.8%
rs62468605	-0.01	0.0065	-0.01	[-0.02; 0.01]	1.3%
rs632766	-0.00	0.0040	-0.00	[-0.01; 0.00]	2.2%
rs6587767	-0.00	0.0039	-0.00	[-0.01; 0.01]	2.2%
rs6782190	0.00	0.0040	0.00	[-0.01; 0.01]	2.2%
rs67999783	-0.00	0.0040	-0.00	[-0.01; 0.01]	2.2%
rs6800021	0.01	0.0040	0.01	[0.01; 0.02]	2.2%
rs710270	0.00	0.0049	0.00	[-0.01; 0.01]	1.8%
rs7157256	0.00	0.0039	0.00	[-0.01; 0.01]	2.2%
rs72687493	-0.00	0.0040	-0.00	[-0.01; 0.01]	2.2%
rs72865608	-0.01	0.0077	-0.01	[-0.02; 0.01]	1.0%
rs7588317	0.02	0.0039	0.02	[0.01; 0.02]	2.2%
rs7646039	-0.01	0.0040	-0.01	[-0.01; 0.00]	2.2%
rs7663288	-0.00	0.0049	-0.00	[-0.01; 0.01]	1.8%
rs7802443	0.00	0.0039	0.00	[0.00; 0.01]	2.2%
rs8000175	0.01	0.0066	0.01	[0.00; 0.02]	1.2%
rs824387	0.01	0.0118	0.01	[-0.02; 0.03]	0.5%
rs9342706	0.01	0.0040	0.01	[0.00; 0.02]	2.2%
rs9356421	0.00	0.0039	0.00	[0.00; 0.01]	2.2%
rs9924529	0.00	0.0050	0.00	[-0.01; 0.01]	1.8%
**Random effects model**			**0.00**	**[0.00; 0.00]**	**100.0%**

Heterogeneity: $I^2 = 49\%$, $\tau^2 < 0.0001$, $p < 0.01$

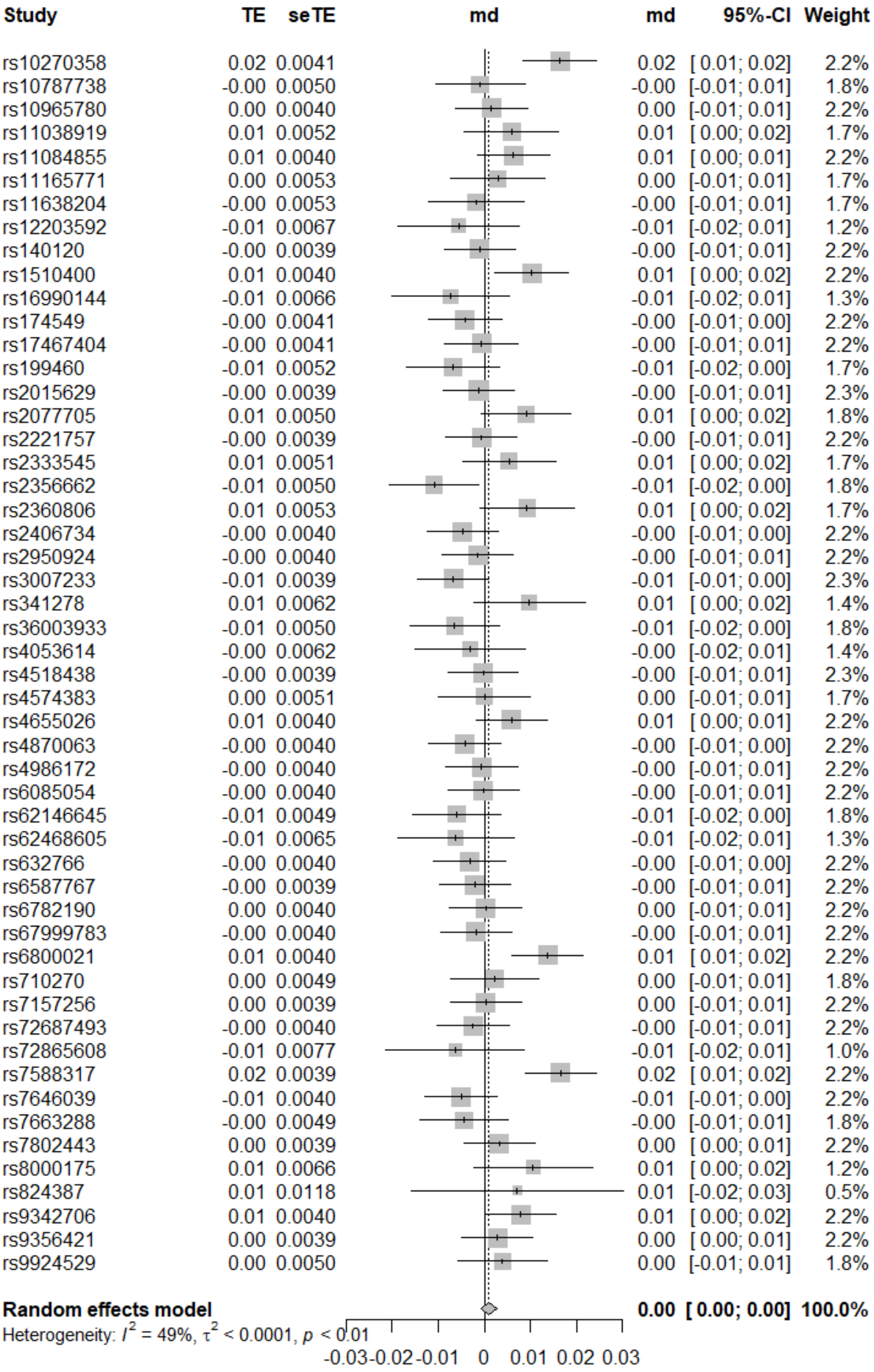

Supplementary Figure S3: Difference in SNP-outcome associations between the UKB and SSGAC when using a 5x10^{-6} p-value threshold.
