# Supplementary figures and images for: Exploring the Lifetime Effect of Children on Wellbeing Using Two-Sample Mendelian Randomisation
Source: Genes (Basel). 2023 Mar 14;14(3):716. doi: 10.3390/genes14030716 (PMC10048211; doi:10.3390/genes14030716)

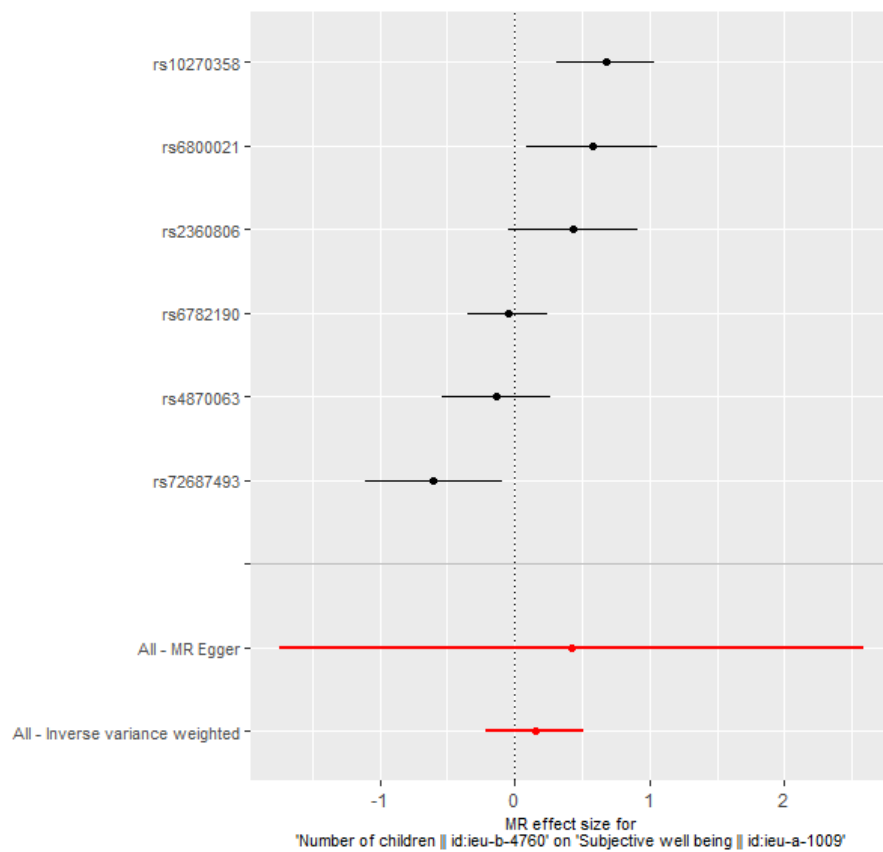

Supplementary Figure S4: Forest plot for the SNP specific Wald ratios for the primary analysis.

Supplement: Supplementary file 1 [file genes-14-00716-s001.zip › cwb Supplementary Figure S4.pdf]

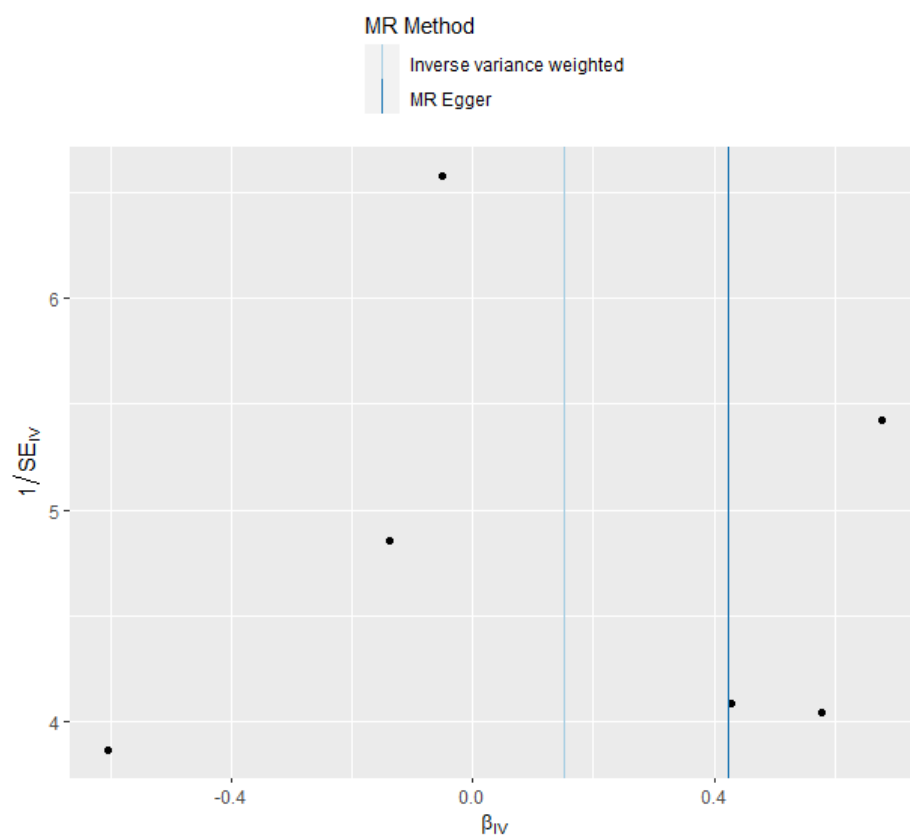

Supplementary Figure S5: Funnel plot for the primary analysis.

Supplement: Supplementary file 1 [file genes-14-00716-s001.zip › cwb Supplementary Figure S5.pdf]

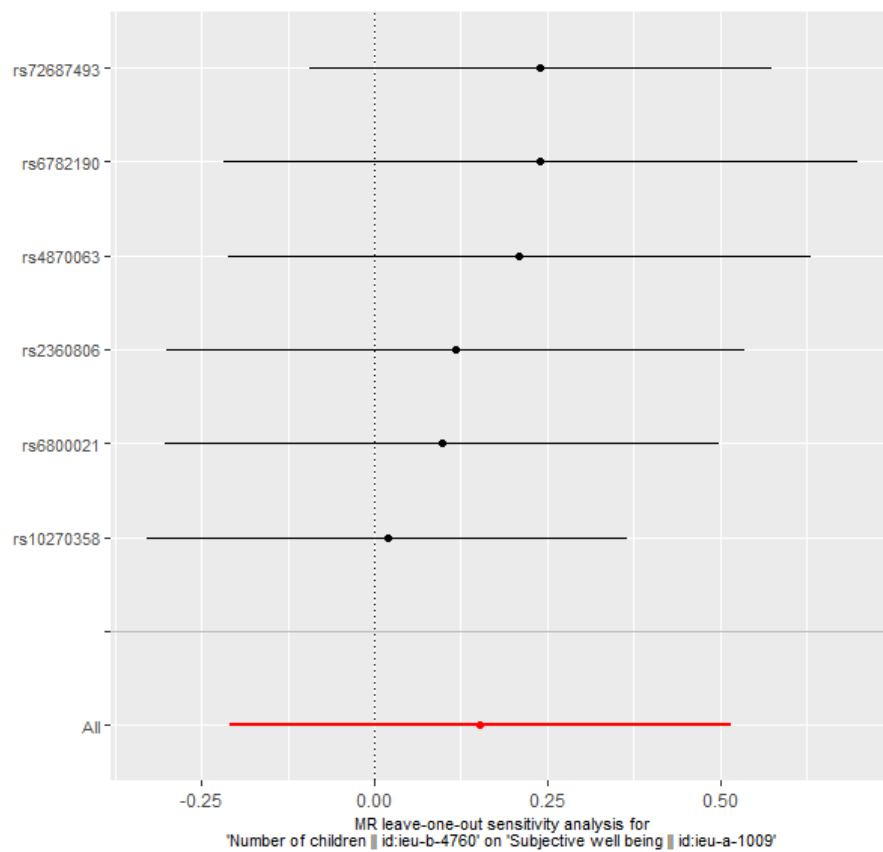

Supplementary Figure S6: Leave-one-out sensitivity analysis.

Supplement: Supplementary file 1 [file genes-14-00716-s001.zip › cwb Supplementary Figure S6.pdf]
